# Annotations

**Published:** 1908-12-05

**Authors:** 


### ANNOTATIONS.

### Corporate Advertising.

Some may remember how that amusing skit, "Signs of the Times," tilted at the Americanisation of newspaper advertisements in this country. The fact is evident enough. In the staidest journals impressions left by information of importance and interest are liable to rude disturbance by the cacophonous drum-beating of the advertisement columns. Editors may certainly reply that the lofty pursuit of advertisement writing is as yet in the state of English literary criticism before Johnson, being without recognised canons. In some cases, however, there may be more material standards, the obligation of which no one can doubt. Just at present there is appearing in the lay Press an appeal to charity on behalf of the St. John's Hospital for Diseases of the Skin, Leicester Square, which reads as follows: "Diphtheria bacilli in the skin of patients have been discovered by our physicians. This form of skin disease is one which has proved most difficult of treatment, and the discovery of a remedy has only recently been made. This remedy has already benefited many who had almost despaired of a cure. We appeal earnestly for funds to enable us to continue our researches and administer our remedies." We are glad to think that the error in tactics this excerpt illustrates is one seldom committed. The authorities of institutions, the medical work of which has chanced to bring them under general notice, have almost always shown the good taste not to allude to the fact in their customary appeals for support. No doubt in the instance under notice there is no breach of professional etiquette; but it is not hard to see that what may be called corporate advertising may prove just as mischievous as the individual variety. A strong sense of solidarity is an excellent thing, except when allowed to override all scruple and delicacy.

### Life Saving Methods.

The relative advantages of the old system of artificial respiration for the apparently drowned known by the late Dr. Silvester's name, and of the new method which has been advocated by Professor Schäfer were commented upon in The Hospital not very long ago (July 11, 1908, p. 379). It appears that the new system has been rapidly gaining adherents, and that as a result of investigations by the Royal Society of Medicine it has been adopted by the Metropolitan police in preference to those of Silvester and Marshall Hall. A demonstration by Professor Schäfer was given at the training home for Metropolitan police recruits a week or two ago, at which magistrates, representatives of every division of the police, and medical men were present. The subsequent vote of thanks was proposed by Mr. Clinton Dent, the chief surgeon of the force, who is convinced of the superiority of the professor's system of resuscitation. It will be remembered that a committee of the Medico-Chirurgical Society has already reported in a similar sense, and that the Royal Life Saving Society has also officially adopted the new method. It is evident, therefore, that it will receive a very thorough and practical trial within the next year or two, and the experiences thus gained will be watched with considerable interest. No radical alteration in treatment based upon theoretical considerations alone, or upon limited practical application, can be allowed to survive unless it establishes a right to do so by the results obtained in actual practice. It is, therefore, important that all those who have facilities for observation of the actual application of the Schäfer system should tabulate carefully the data and publish the conclusions at which they arrive. Police surgeons and constables have unusual opportunities of treating the apparently drowned, and the outcome of official adoption in London will no doubt be a definite pronouncement in a few years upon this important question.

### The General Medical Council.

The recent session of the General Medical Council, which concluded on Saturday last, was noteworthy for at least two reasons. In the first place it marked the fiftieth anniversary of the Council, and in the second place it was brought to a close within the space of five days. The work and progress of the Medical Council during the first fifty years of its existence deserve extended notice, and will be considered at greater length in a future issue. The shortness of its last session is to be attributed, in part at least, to the small number of penal cases brought before the Council. It seemed also to those outside that there was if anything a more businesslike alacrity than usual in the deliberations of this august body. Either of these causes should be a subject for congratulation. The president's opening address was, as usual, interesting and instructive, and was composed in the admirable style which is expected of Sir Donald Macalister. The honours which he and other members of the Council have recently received must be a source of considerable gratification to the Council, for they show that their responsible work and the manner in which it is performed are appreciated in high quarters. The legal status and control of anæsthetic administration and the subject of instruction in anæsthesia in the medical schools were dealt with, though not in any detail, and the regulation of unqualified dentistry was also discussed. On the whole, the medical and dental professions may feel satisfied that these two subjects are being handled by the General Medical Council in as effective a manner as its limited powers and opportunities will permit. The proposed General Anæsthetics Bill, and more especially that part of it which received the provisional approval of the Council, is certainly a much-needed measure and should be productive of great benefit. It will in time restrict general anæsthesia to the hands of those only who have had proper instruction in this as well as other branches of medicine. Unqualified dental practice is a harder problem, and it seems that the Council must continue to deal with it merely in instalments, and not in a sweeping fashion. The drastic methods urged by ardent reformers are unfortunately in the present state of things calculated to do more harm than good.

